# Rivaroxaban: Expanded Role in Cardiovascular Disease Management—A Literature Review

**DOI:** 10.1155/2021/8886210

**Published:** 2021-01-08

**Authors:** Muhammad Ajmal, Jacob Friedman, Qurat Ul Ain Riaz Sipra, Tom Lassar

**Affiliations:** University of Arizona, College of Medicine, Tucson, AZ, USA

## Abstract

Direct oral anticoagulants (DOACs) are widely used for the prevention of stroke in nonvalvular atrial fibrillation, treatment of deep venous thrombosis and pulmonary embolism, and as prophylaxis after hip and knee surgery after approval by the Food and Drug Administration. In the last decade, DOACs were studied for various indications; this review is focused on rivaroxaban, a factor Xa inhibitor, which is used in an expanded evidence-based fashion for coronary artery disease, peripheral artery disease, heart failure, malignancy, and prophylaxis of deep venous thrombosis in acute medical illnesses.

## 1. Introduction

Vitamin K antagonists (VKAs) have been used for anticoagulation in humans since 1954 and were even prescribed to then sitting President Dwight Eisenhower after his myocardial infarction. However, due to various limitations of VKA (e.g., drug-drug interactions, narrow therapeutic window, and need for a blood test to monitor therapeutic effect), DOACs which affect specific targets factor Xa and/or factor II have emerged as a potentially preferred therapeutic strategy to overcome these limitations [1]. Rivaroxaban is a direct factor Xa inhibitor and has been studied in various thromboembolic and atherothrombotic conditions [1]. Factor Xa plays a key role in both intrinsic and extrinsic coagulation pathways leading to the downstream activation of thrombin and clot formation [2]. Rivaroxaban is a small molecule that reversibly inhibits both free and clot-bound factor Xa [2]. It has been used until recently mostly for nonvalvular atrial fibrillation (NVAF) and deep venous thrombosis or venous embolism, but its use is increasing in a growing variety of vascular conditions, notably coronary artery disease, peripheral artery disease, and thromboprophylaxis. The purpose of this review is to provide a comprehensive overview of rivaroxaban use for these rapidly broadening indications.

## 2. Anticoagulation in Nonvalvular Atrial Fibrillation

Atrial fibrillation is one of the most common clinically manifested arrhythmias, causing significant morbidity and mortality due to systemic arterial thromboembolism, particularly stroke [3]. Traditionally, warfarin has been used for anticoagulation in patients with NVAF who require anticoagulation based on the CHA2DS2 or CHA2DS2-VASc score [4]. Because of warfarin's narrow therapeutic window and the need for frequent therapeutic monitoring with the international normalized ratio (INR), DOACs have emerged as a promising anticoagulation strategy for patients with NVAF requiring long-term anticoagulation [5]. Rivaroxaban in the management of NVAF was initially studied in the ROCKET AF trial, which compared rivaroxaban to warfarin for cerebrovascular event prophylaxis and safety. In this trial, 20 mg rivaroxaban daily for patients with creatinine clearance (CrCl) ≥ 50 mL/min or 15 mg rivaroxaban daily in those with CrCl between 30 and 49 mL/min was found to be noninferior to warfarin therapy in reducing thromboembolic events, with a significant reduction in intracranial hemorrhage and fatal bleeding [6]. The results of this trial led to the Food and Drug Administration's (FDA) approval of rivaroxaban in nonvalvular atrial fibrillation on November 4, 2011 [7]. The approval of rivaroxaban various indications in the USA and Europe is shown in Table 1.

The real-world experience based on postmarketing surveillance registry data also showed the safety and efficacy of rivaroxaban in atrial fibrillation patients with comorbidities of diabetes mellitus, chronic kidney disease, acute coronary syndrome, and cancer [8]. Like the ROCKET AF trial, rivaroxaban imparted a significantly lower risk of intracranial hemorrhage in systematic review and metanalysis (rivaroxaban HR, 0.64; 95% CI, 0.47-0.86) compared to warfarin therapy, while providing similar protection against stroke and heart attack [9]. In the systematic review and network meta-analysis comparing DOACs to warfarin, DOACs appear to be at least equivalent to warfarin in preventing stroke in atrial fibrillation with reduced risk of bleeding [10].

In the current American College of Cardiology (ACC)/American Heart Association (AHA)/Heart Rhythm Society (HRS) 2019 focused update on 2014 guidelines, rivaroxaban has a class 1 recommendation, with the level of evidence B in nonvalvular atrial fibrillation with a CHA2DS2-VASc score of 2 or higher in men and three or greater in women [11]. In the 2014 ACC/AHA/HRS guidelines, rivaroxaban, and other DOACs, had a prior class 1 recommendation with the level of evidence B in nonvalvular atrial fibrillation and a CHA2DS2-VASc score of 2 or more in both males and females [12].

## 3. Treatment of Deep Venous Thrombosis and Pulmonary Embolism

Deep venous thrombosis (DVT) is a part of the spectrum which includes superficial thrombophlebitis and pulmonary embolism [13]. After heart attack and stroke, pulmonary embolism (PE) is the third most common cause of death worldwide [14]. Rivaroxaban was initially studied in two dose-finding studies, where a single daily dosage of rivaroxaban was found to be feasible as a treatment for DVT compared to low molecular weight heparin and VKA [15, 16]. These studies led to the EINSTEIN program, consisting of three randomized trials of rivaroxaban. The first trial was for the treatment of acute DVT, the second for the treatment of acute PE, and the third for the continued treatment for those who received treatment for the acute DVT/PE and was continued with rivaroxaban for longer term [17, 18]. The treatment protocol in the acute DVT and acute PE study was rivaroxaban 15 mg twice daily for three weeks followed by 20 mg daily. It was compared to low molecular weight heparin for the median of 8 days until warfarin was therapeutic with an INR level of ≥2. In the continued treatment group, rivaroxaban was compared to the placebo with a dosage of 20 mg daily. In the trials, it was concluded that rivaroxaban might be considered a safe and effective single drug for the treatment of venous thrombosis and venous thromboembolism [16, 17]. This led to the approval of rivaroxaban by the FDA in 2012 [19]. Based on this data of rivaroxaban and the data on other DOACs, the American College of Chest Physicians, in their 10th version of guidelines, recommended to prefer treatment of acute DVT/PE with DOACs over VKAs.

Later, the EINSTEIN CHOICE Investigators studied the 20 mg or 10 mg of rivaroxaban daily and compared it to aspirin 100 m mg daily for extended treatment of venous thromboembolism after 6 or 12 months of initial treatment decided by the treating physicians. In this study, 20 mg or 10 mg daily rivaroxaban was associated with a significantly reduced risk of recurrent venous thromboembolism without an increased risk of bleeding when compared to aspirin [20]. The low dose of rivaroxaban, which is essentially the prophylactic dose, may be considered for longer-term treatment in patients who have already been treated with a full dose to prevent recurrent venous thromboembolism.

Early discharge within 24 hours after low-risk PE was studied in the HoT-PE (The Home Treatment of Patients with Low-Risk Pulmonary Embolism with the Oral Factor Xa Inhibitor Rivaroxaban) trial. Early discharge with rivaroxaban 15 mg twice daily for three weeks followed by 20 mg daily was found to be effective and safe in low-risk acute PE patients [21]. MERCURY PE (Multicenter Trial of Rivaroxaban for Early Discharge of Pulmonary Embolism from the Emergency Department), a randomized, multicenter trial, concluded that early emergency department discharge of low-risk PE patients on rivaroxaban results in significantly lower costs and shorter duration of initial and subsequent hospitalizations without an increase in serious adverse events [22].

The systematic review and network meta-analysis comparing the direct oral anticoagulants found that all DOACs are effective in reducing the risk of venous thromboembolism and preventing death related to venous thromboembolism, although the bleeding profile of apixaban was better compared to other DOACs [23].

## 4. Postoperative Venous Thromboembolism Prophylaxis after Hip and Knee Surgery

Venous thromboembolism (VTE) is a deadly complication after major orthopedic surgeries of hip and knee arthroplasty, and anticoagulants are used to prevent these complications [24]. To prevent VTE, low molecular weight heparin for ten days was used as a standard of care after hip and knee arthroplasty based on the recommendations of the Seventh American College of Chest Physicians recommendations on antithrombotic therapy [25]. This led to additional trials to find oral treatment to prevent VTE complications and associated morbidity and mortality with VTE. Four phase III randomized clinical trials, RECORD 1-4 (Regulation of Coagulation in Orthopedic Surgery to Prevent Deep Venous Thrombosis and Pulmonary Embolism), were conducted to evaluate rivaroxaban compared to enoxaparin. RECORD 1 compared rivaroxaban 10 mg to enoxaparin 40 mg daily for 35 days after hip arthroplasty. RECORD 2 compared rivaroxaban 10 mg daily for 31-39 days to enoxaparin 40 mg for 10-14 days after hip arthroplasty. RECORD 3 compared rivaroxaban 10 mg daily to enoxaparin 40 mg for 10-14 days after knee arthroplasty. Finally, RECORD 4 compared rivaroxaban 10 mg daily for 10-14 days to enoxaparin 30 mg twice daily for 10-14 days [26–29]. All these trials demonstrated the superiority of rivaroxaban over enoxaparin without increasing the risk of bleeding [22–25]. These trials led to FDA approval of rivaroxaban 10 mg daily after hip and knee surgery in 2012 [30]. In the ninth edition of American College of Chest Physicians (ACCP) guidelines, rivaroxaban was recommended to be considered after hip and knee arthroplasty as an alternative to enoxaparin [31].

A prophylactic dose of rivaroxaban was later compared to low-dose aspirin in the EPCAT II (Extended Venous Thromboembolism Prophylaxis Comparing Rivaroxaban to Aspirin Following Total Hip and Knee Arthroplasty II) trial. In this trial, all patients after knee and hip arthroplasty were treated with rivaroxaban for five days and then were divided into two groups. One group received aspirin 81 mg daily, and the second group received rivaroxaban 10 mg daily for nine days after knee replacement and 30 days after hip replacement. After short postoperative prophylaxis of rivaroxaban, aspirin was noninferior to rivaroxaban in preventing venous thromboembolism [32]. The systematic review and meta-analysis of randomized controlled trials evaluating the safety and efficacy of rivaroxaban after total hip and knee replacement found that rivaroxaban is safe and effective with a low incidence of thrombotic and bleeding events [24].

## 5. Venous Thromboembolism Prophylaxis in Acute Medical Illness

Rivaroxaban was studied in the MAGELLAN (Multicenter, Randomized, Parallel-Group Efficacy and Safety for the Prevention of Venous Thromboembolism in Hospitalized Acutely Ill Medical Patients) trial to check the appropriate duration of anticoagulation in these sick patients to prevent VTE [33]. The rivaroxaban group received 10 mg daily dose for 35 ± 4 days with a placebo subcutaneous injection for 10 ± 4 days while the enoxaparin group received 40 mg daily subcutaneous enoxaparin for 10 ± 4 days and an oral placebo for 35 ± 4 days. The efficacy of standard duration rivaroxaban (10 ± 4 days) was like that of enoxaparin, whereas the effectiveness of extended duration (35 ± 4 days) rivaroxaban was superior to enoxaparin. However, rivaroxaban was associated with more negative safety outcomes of clinically relevant bleeding events [31].

Rivaroxaban was also compared to a placebo after hospital discharge for 45 days in the MARINER (Medically Ill Patient Assessment of Rivaroxaban versus Placebo in Reducing Post-Discharge Venous Thrombo-Embolism Risk) trial. However, the results of this trial did not indicate positive health outcomes from the administration of rivaroxaban. Rivaroxaban was not associated with a significantly lower risk of symptomatic VTE and death due to VTE as compared to placebo [34].

Most studies indicated positive health outcomes for those who took rivaroxaban, but some negative and some nonsuperior effects were also found. The FDA approved rivaroxaban to prevent VTE in acutely ill patients in October 2019, and based on this approval, rivaroxaban can be initiated during hospitalizations and continued for 31-39 days [35]. In the systematic review and meta-analysis comparing prolonged thromboprophylaxis with factor Xa inhibitors to the short-term enoxaparin, DOACs were more effective than enoxaparin but were also associated with more bleeding episodes [36].

## 6. Role in Peripheral Artery Disease and Chronic Coronary Artery Disease

Patients with established cardiovascular disease remain at high risk for recurrent cardiovascular events [37]. This led to the landmark COMPASS (Cardiovascular Outcomes for People Using Anticoagulant Strategies) trial, which hypothesized that rivaroxaban with aspirin or alone is more effective than aspirin in preventing recurrent cardiovascular events and is safe in patients with stable atherosclerotic vascular disease [38]. In this randomized trial, it was found that in patients with stable atherosclerotic vascular disease, rivaroxaban 2.5 mg twice daily plus aspirin 100 mg once daily was associated with significantly lower major adverse cardiovascular events (MACE), but significantly higher major bleeding than with aspirin alone. Rivaroxaban 5 mg twice daily alone did not result in a significantly lower risk of MACE but had a significantly higher risk of major bleeding as compared to aspirin alone [34].

Based on this landmark clinical trial, the FDA approved rivaroxaban 2.5 mg twice daily with aspirin 75-100 mg daily in patients with stable coronary artery disease and peripheral artery disease. Although there were more bleeding episodes in the COMPASS trial, there were no intracranial or fatal bleeding events. Based on this, the author is of the opinion that shared decision making should be adopted to prescribe low-dose rivaroxaban 2.5 mg twice daily with aspirin 81 mg daily [39].

In the systematic review and meta-analysis on the use of rivaroxaban in coronary artery disease, it was concluded that patient's characteristics of ischemic and bleeding risk are critical in deciding about the addition of rivaroxaban [40]. The low-dose rivaroxaban in peripheral arterial disease was studied in the systematic review of randomized trials, and it was found to be effective in preventing cardiovascular events with a possible increase risk of bleeding [41].

## 7. Use in Acute Coronary Disease

Patients with acute coronary syndrome manifest increased coagulation system activity in the acute phase which persists even beyond the acute phase [42]. The ATLAS ACS-TIMI 46 (Anti-Xa Therapy to Lower Cardiovascular Events in Addition to Standard Therapy in Subjects with Acute Coronary Syndrome-Thrombolysis in Myocardial Infarction 46) phase II trial was a dose-finding trial. Rivaroxaban 5-20 mg daily total doses in recent acute coronary syndrome were found to reduce major adverse cardiovascular events with dose-dependent increased bleeding events [43]. This led to a phase III trial, ATLAS ACS 2-TIMI 51, which was designed to determine the clinically effective dose of rivaroxaban in recent acute coronary syndrome. In this study, rivaroxaban 2.5 mg twice daily and 5 mg twice daily reduced the primary efficacy endpoint of death from a cardiovascular cause, myocardial infarction, and stroke as compared to placebo. However, there were significantly more adverse events of bleeding with both doses, although the 2.5 dosage had fewer bleeding events than the 5 mg [44]. The direct oral anticoagulants studied in systemic review and meta-analysis were found to have a modest beneficial effect in acute coronary syndrome with an increased risk of bleeding, and shared decision should be made in the use of DOACs in these patients [45].

## 8. Role in Treatment of Venous Thromboembolism with Cancer

VTE is common in patients with cancer, and subcutaneous low molecular weight heparin is the standard therapy in these patients [46]. Therapeutic dose of rivaroxaban was studied in SELECT-D (Anticoagulation Therapy in Selected Cancer Patients at Risk of Recurrence of Venous Thromboembolism), a randomized, multicenter pilot trial, and was compared to the therapeutic dose of dalteparin in patients with active cancer and diagnosis of VTE. There was less cumulative VTE recurrence in the rivaroxaban group compared to the dalteparin group [40]. Based on this trial, it was concluded that rivaroxaban at standard DVT/PE dosage could be considered in patients with active cancer and diagnosis of VTE after shared decision making. The use of DOACs in VTE in cancer patients was found to be as safe and effective as conventional therapy in the systematic review and meta-analysis of 10 studies comparing DOACs with conventional therapy [47].

## 9. Use after Transcatheter Aortic Valve Replacement (TAVR)

In the current 2014 guidelines by ACC/AHA on antithrombotic regimen after transcatheter aortic valve replacement (TAVR), clopidogrel 75 mg daily for the first six months after TAVR has a class IIb recommendation with the level of evidence (LOE) C, along with lifelong aspirin 75 mg daily after TAVR [48]. Because of the reported concern of subclinical TAVR valve thrombosis, rivaroxaban was studied in the GALILEO (Global Study Comparing a Rivaroxaban-based Antithrombotic Strategy after Transcatheter Aortic Valve Replacement to Optimize Clinical Outcomes) trial [49]. This trial compared rivaroxaban 10 mg daily with aspirin 75-100 mg daily in the first three months after TAVR to aspirin 75-100 mg daily with clopidogrel 75 mg daily in the first three months after TAVR. It found that rivaroxaban was associated with higher mortality, thromboembolic complications, and bleeding events compared to the antiplatelet regimen and was terminated early at a median follow-up of 17 months [42]. The use of rivaroxaban is not recommended in prosthetic heart valves. There is also a study being conducted in Brazil called the RIWA (Rivaroxaban vs. Warfarin in Patients with Mechanical Heart Valves) trial. This phase II/III prospective randomized trial is aimed at evaluating the efficacy and safety of rivaroxaban 15 mg twice daily vs. warfarin in mechanical prosthetic valves [50].

## 10. Use in Heart Failure with Reduced Ejection Fraction

Heart failure is considered a hypercoagulable state, and patients with acute or chronic heart failure are at increased risk for thrombotic events, including coronary thrombosis, intraventricular thrombosis, and systemic embolism [51]. The COMMANDER HF (A Study to Assess the Effectiveness and Safety of Rivaroxaban in Reducing the Risk of Death, Myocardial Infarction, or Stroke in Participants with Heart Failure and Coronary Artery Disease Following an Episode of Decompensated Heart Failure) trial was conducted with rivaroxaban 2.5 mg twice daily compared to a placebo in patients with median ejection fraction (EF) of 35% in the rivaroxaban group and 34% in placebo. This was a negative trial: rivaroxaban in patients with recent worsening of chronic heart failure with low EF, and coronary artery disease, was not associated with a reduction in the composite outcome of all-cause mortality, myocardial infarction, or stroke than placebo. Additionally, it did not favorably influence rehospitalization for heart failure [52]. The same outcomes were demonstrated in systematic review and meta-analysis on anticoagulation that rivaroxaban did not reduce mortality, heart failure readmission, or MI in patients with low EF and normal sinus rhythm [53].

## 11. Role in Treatment of Left Ventricular (LV) Thrombus

DOACs have been used off label in LV thrombus, but the data is limited [54]. A three-center cohort study performed on 514 patients with LV thrombus showed that DOACs are associated with higher rates of stroke and systemic embolism than warfarin in a retrospective analysis. It is also interesting to note that patients even with the resolution of LV thrombus on echocardiography still experienced a stroke and systemic embolism [55].

## 12. Use after Left Atrial Appendage Occlusion (LAAO)

LAAO is performed in patients who have nonvalvular atrial fibrillation and cannot take anticoagulants or do not wish to be on anticoagulants [3]. In the landmark clinical trials of LAAO, warfarin was used transiently postoperatively. However, in the real world, there is an increased use of DOACs, but no randomized studies are being conducted on the transient use of DOACs after LAAO [56].

## 13. Antiphospholipid Syndrome (APS)

APS in association with persistent antiphospholipid antibodies of lupus anticoagulant, anticardiolipin antibodies, and/or anti-beta 2 glycoprotein antibodies manifests with arterial and venous thrombosis [57]. Rivaroxaban was studied in thrombotic APS in a randomized noninferiority trial and did not show noninferiority to dose-adjusted warfarin. It also showed a statistically nonsignificant doubling of the recurrent thrombotic events [58].

## 14. Consideration in Kidney Disease

In the ROCKET AF trial, patients with CrCl < 30 mL/min and end-stage renal disease (ESRD) were not included, but rivaroxaban is approved by the FDA with a reduced dose of 15 mg daily with CrCl 15-50 mL/min [6, 7]. This is not recommended in patients with ESRD and atrial fibrillation, and a recent study using Medicare fee-for-service 5% claims data from 2007 to 2013 analyzed treatment and outcomes in patients with atrial fibrillation and ESRD [59]. There was less use of oral anticoagulation in patients with AF and ESRD, and the use of anticoagulation (VKA, apixaban, rivaroxaban, and dabigatran) was not associated with reduced stroke or death but was associated with increased risk of hospitalization for bleeding or intracranial hemorrhage [51]. The use of rivaroxaban in AF was also associated with a higher risk of bleeding in AF patients on hemodialysis in a systemic review of the randomized trials, cohort studies, and case series [60]. Rivaroxaban was not studied in the treatment or prophylaxis of DVT/PE in patients with CrCl < 30 mL/min, while the reduced dose of 2.5 mg twice daily was not tested in patients with CrCl < 15 mL/min [16, 17, 30, 31, 35]. Rivaroxaban is not recommended for the treatment of DVT/PE if CrCl is <30 mL/min, while the 2.5 mg twice daily dose should be used with caution in coronary artery disease and peripheral artery disease with CrCl 15-30 mL/min and is not recommended in ESRD [16, 17, 30, 31, 35]. The dosage of rivaroxaban in various indications and different glomerular filtration rate is shown in Table 2.

## 15. Consideration in Liver Disease

No clinical data is available for the use of rivaroxaban in patients with severe hepatic impairment, and its use is prohibited in Child-Pugh B and C class or any impairment associated with coagulopathy [61].

## 16. Consideration in Obesity

No large randomized controlled trials have specifically investigated the efficacy and safety of DOACs in the obese population [62]. The International Society of Thrombosis and Hemostasis in their recent guidelines of 2016 recommended against the use of DOACs in extremely obese patients with weight > 120 kg or body mass index > 40 kg/m^2^. However, if for some reasons DOACs are used in these patients, then the society recommended checking drug-specific antifactor levels, like antifactor Xa, with the use of rivaroxaban [52].

## 17. Cost Economic Analysis

The cost economic analysis of rivaroxaban for various indications and in different countries is reported in literature. Rivaroxaban is found to be cost-effective and is briefly discussed herein based on the literature search in a few countries. In the Netherlands, rivaroxaban was found to be cost-effective for the treatment and secondary prevention of venous thromboembolism with health gains and cost savings of € 304 over the patient's life compared to LMWH/warfarin [63]. The Brazilian experience also demonstrated cost-effectiveness of rivaroxaban compared to warfarin in the management of venous thromboembolism [64]. Rivaroxaban was also found to be cost-effective in elective electrical cardioversion compared to warfarin in the Netherlands with a 50% probability of being cost-effective compared to warfarin [65]. In Greece, rivaroxaban was also cost-effective in the management of DVT/PE [66]. Rivaroxaban was also found to cost-effective in the treatment of venous thromboembolism in the Chinese population when compared to warfarin [67]. In the elderly population in the US with worsening renal function, rivaroxaban was found to be cost-effective compared to warfarin for the treatment of nonvalvular atrial fibrillation [68]. In the treatment of PAD when clinic-economic analysis of rivaroxaban compared to clopidogrel was performed, rivaroxaban was found to be cost-effective with a saving of 682 US dollars per participant [39]. Rivaroxaban was found to be cost-effective in the management of cancer-associated thrombosis when compared to dalteparin in the US population [69]. The cost of stable atherosclerotic disease was analyzed in the Australian population; rivaroxaban with aspirin was found to be cost-effective compared to aspirin alone [70]. Rivaroxaban was also found to be cost-effective compared to enoxaparin for the prevention of venous thromboembolism after hip and knee replacement surgeries [71].

## 18. Safety

Bleeding is one of the main side effects of rivaroxaban, and it can vary from minor bruising to major life-threatening bleeding. Intracranial, gastrointestinal, and other intracavitary bleeding events are reported with rivaroxaban, although less frequently than with warfarin [72]. Based on the systematic review and metanalysis of randomized trials comparing the risk of gastrointestinal bleeding in patients taking direct oral anticoagulants, rivaroxaban and dabigatran were associated with increased gastrointestinal bleeding compared to other direct oral anticoagulants [73]. These bleeding events usually occur in patients with other comorbid conditions like hypertension, abnormal liver and renal functions, old age, concomitant use of other blood thinners, and previous episodes of bleeding [74].

Another potential limitation is premature discontinuation of this drug as it is associated with higher risk of thrombosis and is strongly discouraged because of this reason [75, 76]. As rivaroxaban can increase bleeding tendency in various invasive procedures, although there are no definite guidelines, rivaroxaban should be stopped for about two half-lives before spinal puncture/anesthesia to prevent spinal hematoma [75, 76].

Rivaroxaban has several drug interactions and should not be used with P-glycoprotein and CYP3A inhibitors or inducers because of the variability in the efficacy of rivaroxaban and increased risk of thrombosis or bleeding [77].

Liver injury, hypersensitivity reactions, leukocytoclastic vasculitis, and hair loss are nonhemorrhagic but rare side effects of rivaroxaban reported in literature [78].

## 19. Rivaroxaban Reversal Agent

The factor Xa reversal, andexanet alpha, which is a modified recombinant inactive form of human factor Xa, was studied in ANNEXA-4 (Andexanet Alpha, A Novel Antidote to the Anticoagulation Effects of Factor Xa Inhibitors), a single group cohort study [79]. In this study, it was found that in patients with major acute bleeding associated with the use of factor Xa inhibitor, treatment with andexanet significantly reduced factor Xa activity, and 82% of the patients had an excellent or good hemostatic efficacy at 12 hours. Andexanet alpha was approved by the FDA in May 2018 [80]. It is available in low and high doses. A low dose is 400 mg intravenous bolus at the rate of 30 mg/minute followed by 4 mg/hour for 2 hours for patients taking ≤10 mg daily rivaroxaban or if >10 mg daily dose with the last dose taken >8 hours before the administration of andexanet. The high dose is 800 mg intravenous bolus at the rate of 30 mg/minute followed by 8 mg/hour for 2 hours for rivaroxaban dose > 10 mg with the last dose hours before the administration of andexanet < 8 hours.

## 20. Conclusion

Rivaroxaban has been studied in various indications and has robust positive data in numerous cardiovascular indications, including nonvalvular atrial fibrillation, treatment, and prophylaxis of deep venous thrombosis and pulmonary embolism, peripheral artery disease, stable coronary artery disease, acute coronary syndrome, and venous thromboembolism in cancer patients. This data is supported by observational studies, randomized trials, systematic review, and meta-analysis. Rivaroxaban is also proved to be cost-effective worldwide compared to standard of care therapies in various indications.

On the other hand, the randomized clinical trials of rivaroxaban in the transcatheter aortic valve, thrombotic antiphospholipid syndrome, and heart failure with reduced ejection fraction and observational cohort study in left ventricular thrombus were negative, and rivaroxaban should not be used in these patients.

## Figures and Tables

**Table 1:** Indications and approval of rivaroxaban.

Indication	Year of approval	Country
DVT/PE prophylaxis in hip and knee surgery	2008 in Europe 2011 in USA	
Atrial fibrillation	2011	USA and Europe
DVT/PE treatment	2012	USA and Europe
Acute coronary syndrome	2013	Europe
To reduce risk of VTE after 6 months of treatment of DVT/PE	2017	USA and Europe
Stable CAD	2018	USA and Europe
PAD	2018	USA and Europe
DVT/PE prophylaxis in acute medical illness	2019	USA

DVT/PE: deep vein thrombosis/pulmonary embolism; CAD: coronary artery disease; PAD: peripheral arterial disease.

**Table 2:** Rivaroxaban dosage in various indications.

Indication	GFR	Dose
Nonvalvular AFib	>50 mL/min	20 mg daily
	15-50 mL/min	15 mg daily
	<15 mL/min	Not recommended

Treatment of DVT/PE	>30 mL/min	15 mg BID for 3 weeks followed by 20 mg daily.
	<30 mL/min	Not recommended

DVT/PE prophylaxis in hip and knee surgery	>30 mL/min	10 mg daily
	<30 mL/min	Not recommended

DVT/PE prophylaxis in acute medical illness	>30 mL/min	10 mg daily
	<30 mL/min	Not recommended

Stable coronary artery disease	>30 mL/min	2.5 mg BID
	15-30 mL/min	2.5 mg BID with caution
	<15 mL/min	Not recommended

Peripheral artery disease	>30 mL/min	2.5 mg BID
	15-30 mL/min	2.5 mg BID with caution
	<15 mL/min	Not recommended

Acute coronary artery syndrome	>30 mL/min	2.5 BID
	15-30 mL/min	2.5 mg BID with caution
	<15 mL/min	Not recommended

GFR: glomerular filtration rate; BID: twice daily; AFib: atrial fibrillation; DVT/PE: deep vein thrombosis/pulmonary embolism.
